# Complete response to capmatinib in a patient with metastatic lung adenocarcinoma harboring *CD47-MET* fusion: a case report

**DOI:** 10.1093/oncolo/oyae106

**Published:** 2024-06-04

**Authors:** Glaucia Alves de Souza, Debora Maciel Santana Dornellas, Paulo Vidal Campregher, Carlos Henrique Andrade Teixeira, Gustavo Schvartsman

**Affiliations:** Hospital Israelita Albert Einstein, São Paulo, Brazil; Hospital Israelita Albert Einstein, São Paulo, Brazil; Hospital Israelita Albert Einstein, São Paulo, Brazil; Hospital Alemão Oswaldo Cruz, São Paulo, Brazil; Hospital Israelita Albert Einstein, São Paulo, Brazil

**Keywords:** *MET* receptor tyrosine kinase, non-small cell lung cancer, capmatinib, next-generation sequencing, case report

## Abstract

Comprehensive genomic profiling is highly recommended for treatment decision in nonsquamous, non-small cell lung cancer (NSCLC). However, rare genomic alterations are still being unveiled, with scarce data to guide therapy. Herein, we describe the treatment journey of a 56-year-old, never-smoker Caucasian woman with a metastatic NSCLC harboring a *CD47-MET* fusion, initially classified as a variant of unknown significance. She had undergone 3 lines of therapy over the course of 3 years, including chemotherapy, immunotherapy, and anti-angiogenic therapy. After reanalysis of her next-generation sequencing data in our service, the fusion was reclassified as likely oncogenic. The patient was started with fourth-line capmatinib, with a good tolerance so far and a complete metabolic response in the active sites of disease, currently ongoing for 18 months. In conclusion, we highlight the sensitivity of a novel *MET* fusion to capmatinib and emphasize the need for comprehensive panels in NSCLC and molecular tumor board discussions with specialized centers when rare findings arise.

## Teaser

This case report highlights the sensitivity of a novel MET fusion to capmatinib and emphasizes the need for comprehensive panels in non-small cell lung cancer and molecular tumor board discussions with specialized centers when rare findings arise.

**Key Points**

- Next-generation sequencing platforms have become widely used to identify driver alterations in patients with non-small cell lung cancer (NSCLC). However, with the increasing use of comprehensive panels, rare molecular findings have come to light.
- *MET* fusions are infrequent types of structural rearrangement reported in 0.26% of NSCLCs. Often, novel fusion partners are discovered, with scarce data to guide therapeutic decisions for these patients, since guidelines for gene fusion curation are not yet available.
- Herein, we report a case of a lung adenocarcinoma in a nonsmoking woman, who was found to have a rare CD47-MET fusion, which was initially classified in 2020 as a variant of unknown significance by an outside facility, and later reviewed by our bioinformatics team as likely oncogenic. The patient received fourth-line capmatinib and had a complete metabolic response, ongoing for 18 months.

## Introduction

The diagnosis and therapeutic approach to non-small cell lung cancer (NSCLC) are undergoing major changes, especially in recent years with the development of new targeted therapy agents, immunotherapy, and antibody-drug conjugates. Therefore, comprehensive genomic profiling is highly recommended for treatment decisions among nonsquamous NSCLC, particularly in women with no/light smoking history at a younger age.

However, with the increasing use of comprehensive panels, rare molecular findings have come to light.^1^*MET* gene aberrations are now well described, with *MET* exon 14 skipping being the most common, occurring in 2%-4% of NSCLC and predicting sensitivity to *MET* inhibitors such as crizotinib, capmatinib, tepotinib, and savolitinib. *MET* amplifications occur in 1%-5%, but infrequently represent driver alterations, rather being an adaptive mechanism of resistance to therapies.^2^*MET* fusions are infrequent types of structural rearrangement reported in 0.26% of NSCLCs.^3^ Often, novel fusion partners are discovered, with insufficient data to guide therapeutic decisions for these patients, since guidelines for gene fusion curation are not yet available.

Herein, we describe the treatment journey of a nonsmoking woman diagnosed with lung adenocarcinoma, who was found to have a rare *CD47-MET* fusion and achieved an excellent response with capmatinib.

### Patient story

In September 2019, a 56-year-old, never-smoker female patient was diagnosed with a lung adenocarcinoma arising in the lower lobe of the left lung, with liver and abdominal lymph node metastases. No brain lesions were found. Immunohistochemistry for PD-L1 showed a tumor proportion score of 30%. After DNA and RNA extraction from FFPE tumor tissue, the samples were sequenced using Trusight Tumor 170 (Illumina) in an outside facility. This assay detects single nucleotide variations, small indels, and copy number variations through DNA analysis and gene fusions with RNA sequencing. The following variants, classified at the time as variants of unknown significance (VUS), were detected by DNA/RNA sequencing: *MET-CD47* fusion, *FGF3* (NM_005247.2) c.133C>A p.(Arg45Ser); *MAP2K2* (NM_030662.3) c.814G>A p.(Ala272Thr); *PDGFRA* (NM_006206.5) c.1673G>A p.(Arg558His); *RET* (NM_020975.4) c.3217_3218dupAG p.(Ser1073ArgfsTer37); and *ROS1* (NM_002944.2) c.6985C>G p.(Pro2329Ala).

Based on these findings, a nontargeted treatment approach was taken, between 2019 and 2022, during which the patient received first line with carboplatin, pemetrexed, and pembrolizumab, second line with docetaxel and ramucirumab, and a third line with carboplatin and gemcitabine, eventually with disease progression in all sites of disease.

**Figure 1.**
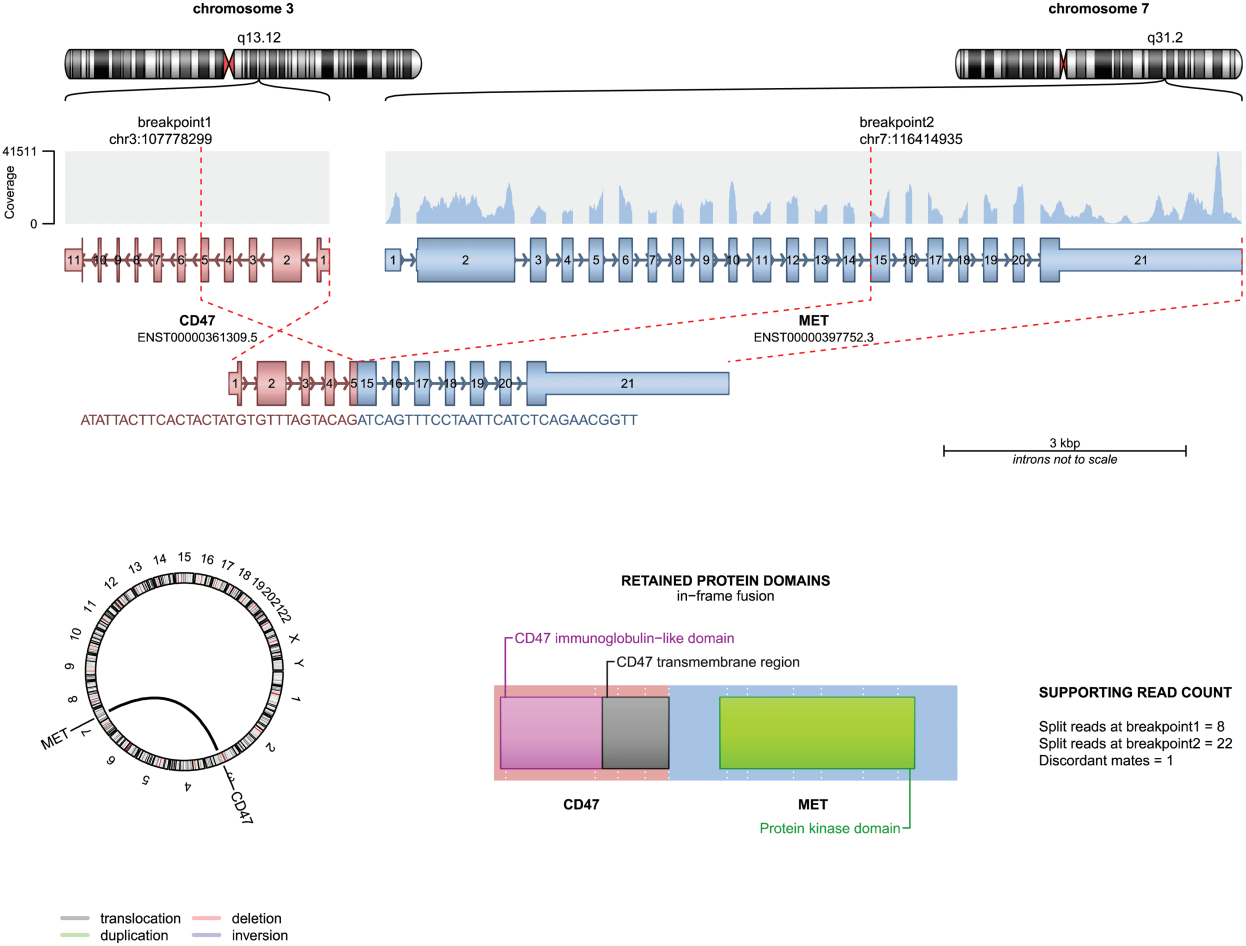
Schematic representation of CD47::MET gene fusion (Arriba output).

At that moment, the patient sought a second opinion at our hospital. The RNA sequencing data were reprocessed in our center using an in-house bioinformatics pipeline in association with the use of the gene fusion callers manta (Illumina) and arriba.^4^ We detected a gene fusion between *CD47* (exon 5) and *MET* (exon 15) (Figure 1). Based on software reconstruction of the protein after DNA/RNA sequencing, the fusion breakpoint was intronic and the MET kinase domain was intact, leading the bioinformatics analyst to classify it as likely oncogenic, rather than a variant of unknown significance. Shortly afterward, a case report of a patient harboring the same fusion demonstrating a clinical response to the kinase inhibitor crizotinib corroborated that interpretation, which aided the process to obtain insurance approval for a MET inhibitor to the patient.^5^

**Figure 2.**
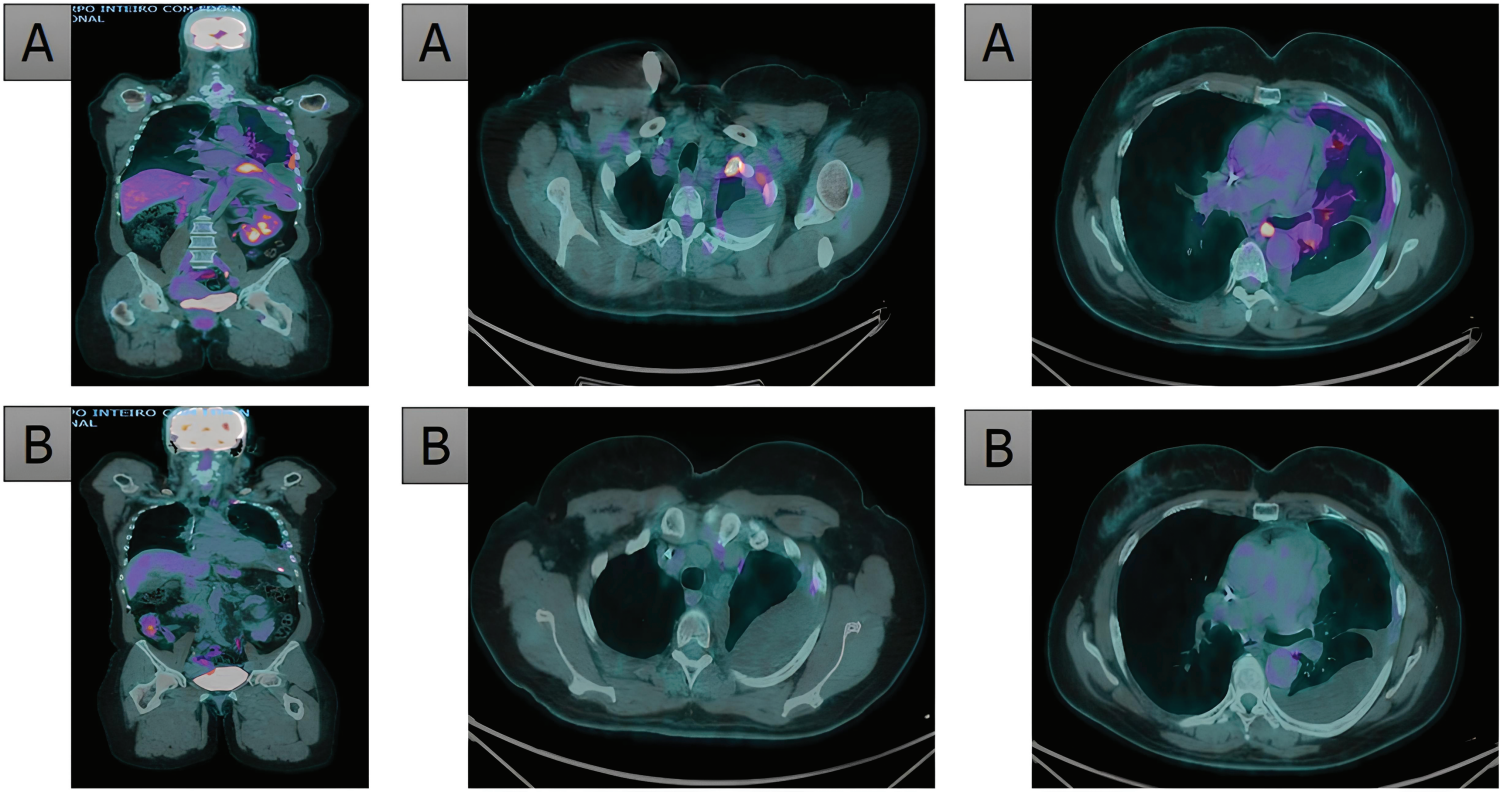
Tumor response to capmatinib treatment. A: Baseline before treatment; B: 6 months after capmatinib.

After discussion of risks and benefits, the patient agreed to start capmatinib, opted due to its more potent activity against the MET tyrosine kinase domain and better safety profile when compared to crizotinib.^6^ She has been on therapy for 18 months, with no significant adverse events. A restaging PET-CT revealed a complete metabolic response in all sites of active disease, a response which is currently ongoing (Figure 2).

### Molecular tumor board

We present a rare finding in a nonsmoker female patient with NSCLC harboring a *CD47-MET* fusion that responded to capmatinib. Through this report, we emphasize 2 important messages: first, that comprehensive, hybrid capture-based genomic profiling is an indispensable part of lung cancer investigation, particularly for patients with a high epidemiological probability of harboring a driver alteration (females, non/light-smokers, and younger age). The hybrid capture method with RNA sequencing allows for the detection of both known and novel fusion partners. Second, rare findings, often previously unreported, should be discussed in molecular tumor boards with cancer genomics experts to evaluate the potential oncogenicity of molecular abnormalities in the absence of robust literature.

The lack of specific guidelines for the curation of rare and undocumented gene fusions continues to present a challenge for molecular pathologists and oncologists alike. While gene fusions represent common oncogenic driver events in many tumor types, the presence of nononcogenic gene fusions (passenger events) is also frequent in cancer.^7^ The gold-standard method to determine the oncogenicity of a fusion involves plasmid construction containing the fusion sequence, transduction within cultured cells, and downstream signaling detection. Additionally, drug sensitivity tests can be performed to assess which available or experimental drugs are capable of binding and blocking its activity.^8^ Naturally, this process requires not only significant financial and human resources, but also time—which patients with refractory metastatic tumors do not have. Therefore, fusion protein reconstruction by software may be a surrogate to identify oncogenic characteristics, such as whether the fusion is in frame and if the kinase domain of the target gene is intact. Oncogenic *MET* gene fusions occur only in about 0.3% of lung cancer patients and have been reported with multiple gene partners, such as *CAV1*, *CD74*, *KIF5B*, and *HLA-DRB1*, among others.^9^ In these oncogenic fusions, *MET* is typically the 3ʹ partner, leading to in-frame fusions with preserved kinase domains. In the present case, the *CD47-MET* fusion was initially classified as a VUS possibly due to the lack of a previous description in the medical literature. Moreover, it was inaccurately described in the report as a *MET-CD47* fusion, against the usual reporting of 5ʹ-to-3ʹ gene orientation.^10^ These aspects highlight the need for training of bioinformatics experts in addition to sequencing hardware acquisition, as data curation is just as relevant as the sequencing library itself.

Knowledge of the spectrum of activity, safety, and potency of available drugs is also relevant. Crizotinib was used in the single case report presented so far where the *CD47-MET* fusion was found. However, due to its activity against other proteins, such as *ROS1* and *ALK*, its potency against MET is lower and toxicity more pronounced. Since the *MET* kinase domain was intact in the present fusion, the treatment selection could be broadened to all drugs available with activity against *MET* kinase, with efficacy expected to improve by utilizing novel, more specific, and potent agents such as capmatinib or tepotinib.

In conclusion, we discuss a patient’s journey after detecting a rare *CD47-MET* fusion and describe a durable complete metabolic response with capmatinib. This report provides support for the off-label use of medications in clinical practice with paucity of data until protein function characterization is performed.

## Data Availability

The data underlying this article are available in the article.
